# A comparison of lumbar transverse pedicle angles between ethnic groups: a retrospective review

**DOI:** 10.1186/s12891-019-2507-2

**Published:** 2019-03-18

**Authors:** Robert Stockton, Joseph Albano, Jonathon Lentz, Maximillian Ganz, Kanwarpaul Grewal, Gus Katsigiorgis

**Affiliations:** 1 Department of Orthopedic Surgery, Northwell Health Plainview Hospital, 888 Old country road, Plainview, NY 11803 USA; 2 NYIT College of Osteopathic Medicine, Old Westbury, NY USA

**Keywords:** Transverse pedicle angle, Spinal fusion, Spine surgery, Pedicle, Computerized tomography, CT

## Abstract

**Background:**

Spinal surgery requires an intimate understanding of pedicle morphology to provide safe and effective outcomes. Although current research has attempted to identify morphological vertebral pedicle trends, no study has utilized computed tomography (CT) scans to compare the lumbar transverse pedicle angle (TPA) with patient demographics factors in a diverse population throughout multiple hospital centers.

**Methods:**

Analysis of randomly selected CT scans from L1-L5 of 97 individuals who underwent imaging over a two-week period for non-back pain related complaints was conducted. Measuring 9**7**0 TPAs in total allowed for comparison of each patients’ pedicle angle with important patient specific demographics including ethnicity, age, gender, height and weight. Statistical analysis utilized multiple comparisons of demographics at each level with post-hoc Bonferroni correction analysis to compare demographics at each level.

**Results:**

With relation to gender, age, height or weight, no statistically significant differences were identified for TPAs at any vertebral level. However, when stratified by ethnicity, the differences in transverse pedicle angles averages (TPA –Avg) at L2 and L3 were found to be statistically significant (*p* < 0.05).

**Conclusion:**

We have identified a previously unknown and significant relationship between ethnicity and TPA at lumbar vertebral levels. These findings provide critical information that may be added to the operating surgeons’ knowledge of pedicle morphology. We hope this novel information can assist in preoperative planning of pedicle screw placement and potentially help improve surgical outcomes.

## Background

Posterior lumbar fusion has been utilized to alleviate pain and instability in patients with spinal injuries and deformities and for nearly 65 years [1]. When lumbar fusion is indicated, there are multiple different techniques utilized to achieve fusion [2, 3]. With advancing trends and technologies in surgery, there is an increased impetus to advance patient outcomes by improving operative techniques and lumbar fusion is no exception [4, 5]. With the increasing use of posterior lumbar fusion, further elucidation of patient specific variables in relation to vertebral morphometric variation may assist orthopedic surgeons in planning and performance of spinal surgery.

**Fig. 1:**
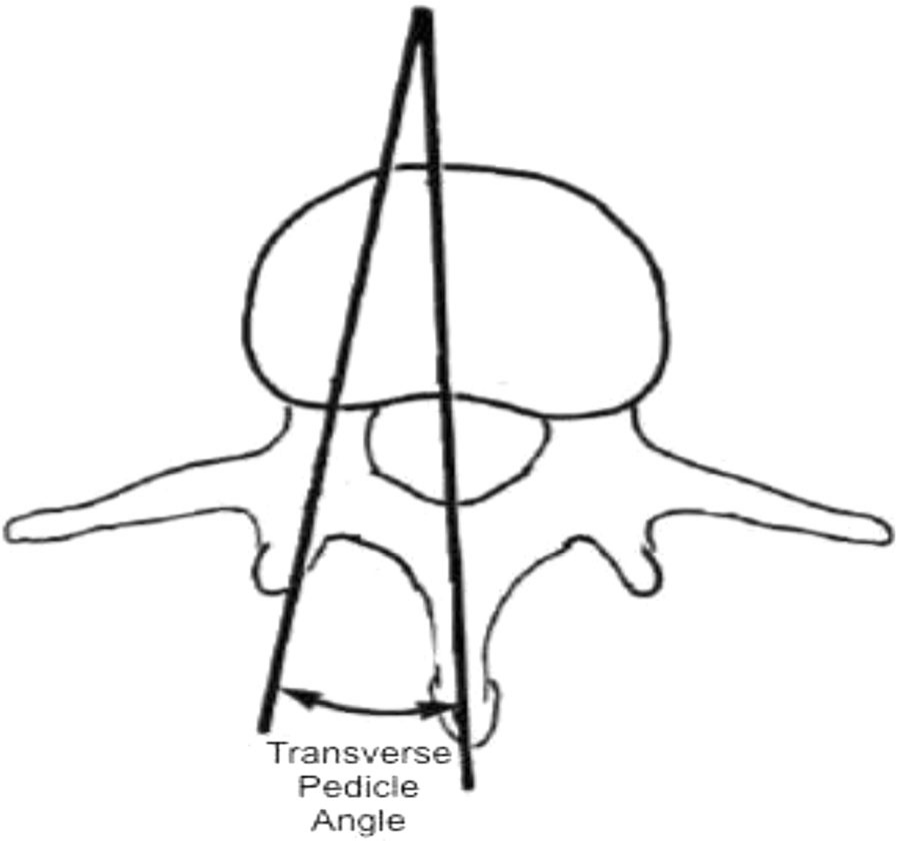
TPA Measurement. Each lumbar TPA was measured by creating a midline measurement from spinous process to the anterior vertebral body then measuring the angle from that first central line to the mid-axis of the pedicle bilaterally

An understanding of the osseous vertebral anatomy and variation between patients is of the utmost importance in spinal fusion, and even more so in with the increasing popularity of minimally invasive spinal fusion as the surgeon may have less visual reference available. Slight deviation in screw trajectory could have devastating outcomes for patients. A better understanding of anatomic variability offers to improve patient safety by increasing the surgeons’ precision while performing spinal fusion procedures. Specifically, the transverse pedicle angle (TPA) is utilized by the operating physician to gauge optimal course of pedicle screw placement. The TPA is the angle created between a line drawn from the midline of the spinous process to the anterior vertebral body and the mid-axis of the pedicle (Fig. 1). Identification of this angle is key for guidance of pedicle screw trajectory. Orthopedic surgical textbooks suggest that knowledge of this angle is important for ideal insertion of pedicle screws from the posterior aspect [6]. There is currently a lack of knowledge regarding patient factors that may influence the morphology of the TPA, and thereby the ideal screw trajectory for successful spinal fusion. Utilization of computed tomography (CT) to identify TPA angle in each patient at each vertebral level of fusion is ideal, however, most surgeons do not use this modality to assist planning surgery and may not have this option in emergent situations.

Multiple studies have attempted to utilize diverse modalities to help outline common anatomical parameters of vertebrae and thus help surgeons when planning spinal surgery. Morales-Avalos, et al. [7] have displayed significant correlation in thoracic pedicle variability with age and gender utilizing caliper measurements on dried osseous specimens from a Mexican population. Yu, et al. [8], have shown significant correlation between lumbar pedicle morphometry, gender, height and weight utilizing digital calipers on a population of American human cadavers. Gulec, et al. [9], have utilized three-dimensional CT to compare gender, age and height with pedicle morphometry in a Turkish only population. Another study utilized electronic calipers to compare the pedicles in a small (12 specimen) Greek only cadaveric study [10]. A study utilizing CT reported lumbar pedicle morphometry in a population of Pakistani patients only [11]. Mughir, et al. [12], compared pedicle morphology between adults and children in a Malaysian population. In a study of patients with low back problems, lumbar spine morphometry was compared to patient gender on CT [13]. Chadha, et al. [14], observed multiple pedicle characteristics in an Indian only population. The authors of that study then reviewed previous literature on TPAs and noted that Indians have different TPAs at some vertebral levels when compared to Western populations. However, this comparison was made between multiple studies utilizing multiple and differing measurement techniques opening the possibility for unreliable correlations. A more recent study undertaken in South Africa has attempted to identify ethnic variation in osseous morphology utilizing 174 dried lumbar vertebral specimens with caliper and goniometer measurements [15]. A meta-analysis study regarding CT analysis of the osseous morphology in the cervical spine undertaken by Marumo, et al. [16], claims that although there is variation due to ethnicity, there may be a lack of significance.

Still, none of these aforementioned studies provide a large-scale, generalizable and reliable database of CT imaging and measurement of pedicle attributes. A single study utilizing homogenous measurement techniques among a diverse living population without reported back pain is necessary to delineate variations in pedicle morphology related specifically to ethnicity, age, gender, height, and weight. When instrumenting for lumbar fusion, a thorough understanding of the vertebral TPA is integral in safely and precisely placing pedicle screws in the lumbar spine. To our knowledge there has never been a single study of measuring TPA in living adults using CT scan that includes multiple different races and ethnicities.

The specific aim of this research was to create a single study comparing the TPAs of patients in a diverse area of the country. This population allows for analysis of potential trends between multiple ethnicities and other demographic characteristics under the same measurement methodology in order to identify if there are any significant differences in TPA among races.

## Methods

A retrospective review of CT scans of the abdomen and pelvis was performed over a two-week period (between July 1, 2016 and July 14, 2016). The CT scans were performed at seven hospitals within one single health system. We randomly selected 97 CT images of L1-L5 from all scans completed during this time period. Using the CT abdomen and pelvis studies rather than lumbar spine specific CT scans allowed for screening of a population of 9**7** patients who presented with chief complaints unrelated to back pain. CT scans were reviewed on Carestream PACS and the present “Bone Window” was utilized for evaluation and analysis of the CT scans. From each of the randomly selected CT scans the TPA from L1-L5 were measured. In total, 9**7**0 lumbar TPA’s were evaluated.

Each lumbar TPA was measured by creating a midline measurement from spinous process to the anterior vertebral body and measuring the angle from that midline to the mid-axis of the pedicle bilaterally (Fig. 1). TPA data was obtained by a single observer and verified by 2 more senior physicians, enhancing interobserver variations. We then compared TPA with multiple patient factors including ethnicity, age, gender, height and weight. Height and weight were directly measured and reported in patient charts. Inclusion criteria for age was 18 through 99 years. Analysis was carried out by a Senior Research Statistics Analyst to determine the significance of the study findings.

Those excluded from the study were patients with evidence of prior lumbar spine surgery on imaging, scans that did not allow analysis of the five lumbar segments and patients with evidence of scoliosis.

## Results

**Fig. 2:**
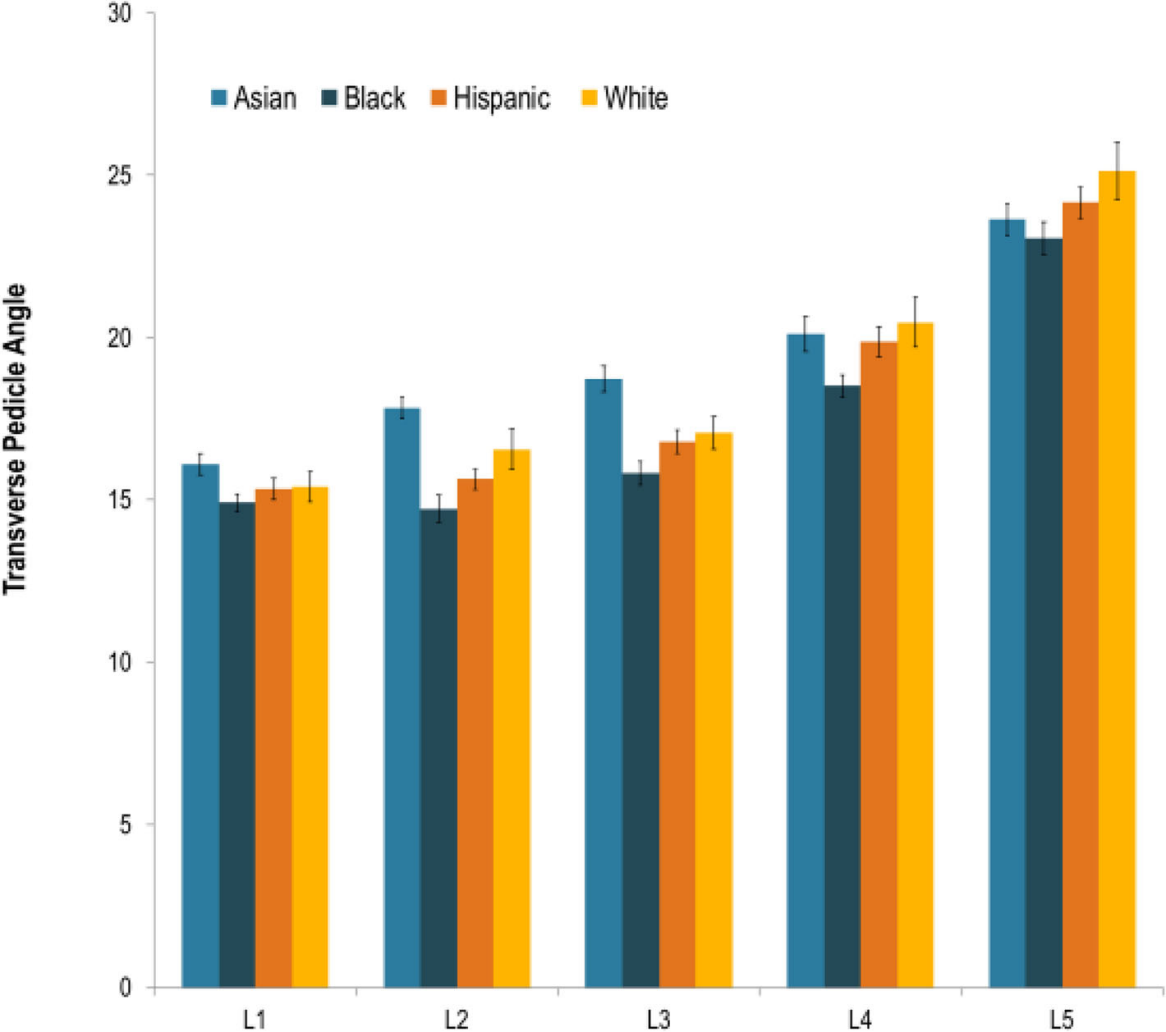
TPA Separated by Race at Lumbar Segments L1-L5. This graph depicts the differences seen between race at each lumbar level with respect to mean TPA and race. Error bars indicate standard error of the mean (SEM) for each category

The ethnicities of the patients from which we obtained these scans were: Asian (*n* = 31), Hispanic (*n* = 27), Black (*n* = 27), and White (*n* = 12). TPA mean, standard deviation (SD) and standard error of the mean (SEM) with respect to each lumbar segment and ethnicity are reported (Table 1). In all ethnicities an increase in the TPA was appreciated with progression down each lumbar segment from L1 to L5. For each individual lumbar segment, the TPA mean with respect to ethnicity was reported with each corresponding standard error and is displayed graphically (Fig. 2).

**Table 1:**
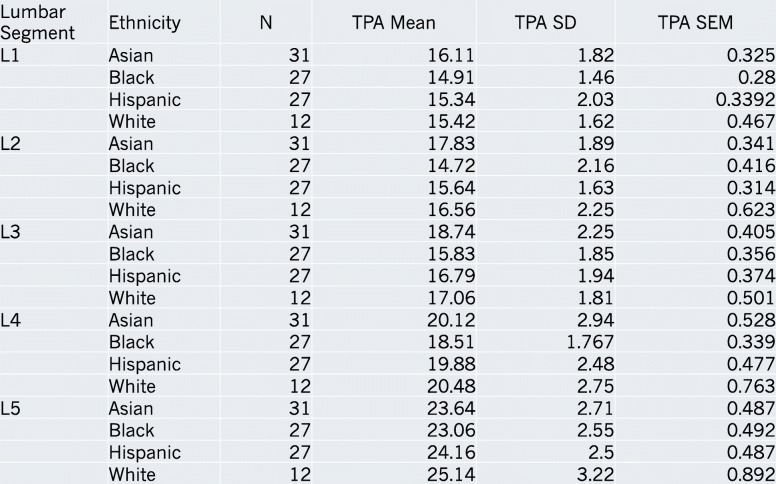
Mean TPA Data. Data obtained via CT analysis of mean TPA, standard deviation and standard error of the mean classified by race and individual lumbar level

When statistically analyzing TPA with other variables, there were no statistically significant differences found for TPA with relation to gender, age, height or weight. However, when stratified by ethnicity, the TPA averages at individual vertebral levels were found to be statistically significant at the L2 and L3 levels (*p* < 0.05). No statistically significant findings were found at levels L1, L4, or L5.

At L2, Asians had a mean TPA-Avg angle of 17.83^o^, Whites had a mean TPA-Avg angle of 16.56^o^, Hispanics had a mean TPA-Avg angle of 15.34^o^, and Blacks had a mean TPA-Avg angle of 14.91^o^. At L3, Asians had a mean TPA-Avg angle of 18.74^o^, Whites had a mean TPA-Avg angle of 17.06^o^, Hispanics had a mean TPA-Avg angle of 16.79^o^, and Blacks had a mean TPA-Avg angle of 15.83^o^.

Multiple comparisons between ethnicities at each level were made followed by post-hoc comparisons utilizing Bonferroni correction indicated that there is statistical significance at L2 and L3 at the level *p* < 0.05 (Table 2). When multiple comparisons were made at L2, Asians were found to have significantly larger TPA-Avg angle by 3.11^o^ (*p* < 0.0001) when compared to Blacks. Additionally, at L2, the TPA-Avg angle of White individuals is 1.84^o^ larger than black individuals, which was found to be significant (*p* = 0.039). When multiple comparisons were made at L3, Asians were found to have a TPA-Avg of 1.94^o^ (*p* = 0.002) and 2.91^o^ (*p* < 0.0001) larger than Hispanics and Blacks respectively.

**Table 2:** Multiple Comparisons. Post-Hoc Bonferroni analysis of multiple comparison for each ethnicity at each individual vertebral level was undergone to identify significance

Multiple Comparisons							
Lumbar Level	Ethnicity	Ethnicity Comparison	Mean Difference	SEM	Significance	95% Confidence Interval	
						Lower Bound	Upper Bound
L1 TPA- Avg	White	Asian	−.686680	.600579	1.000	− 2.30576	.93240
		Hispanic	.080972	.612868	1.000	−1.57124	1.73318
		Black	.510787	.612868	1.000	−1.14142	2.16300
	Asian	White	.686680	.600579	1.000	−.93240	2.30576
		Hispanic	.767652	.465006	.613	−.48594	2.02125
		Black	1.197467	.465006	.070	−.05613	2.45106
	Hispanic	White	−.080972	.612868	1.000	−1.73318	1.57124
		Asian	−.767652	.465006	.613	−2.02125	.48594
		Black	.429815	.480773	1.000	−.86628	1.72591
	Black	White	−.510787	.612868	1.000	−2.16300	1.14142
		Asian	−1.197467	.465006	.070	−2.45106	.05613
		Hispanic	−.429815	.480773	1.000	−1.72591	.86628
L2 TPA- Avg	White	Asian	−1.274615	.645813	.308	−3.01524	.46600
		Hispanic	.918348	.659796	1.000	−.85996	2.69665
		Black	1.835014^a^	.659796	.039	.05671	3.61332
	Asian	White	1.274615	.645813	.308	−.46600	3.01524
		Hispanic	2.192963^a^	.514499	.000	.80627	3.57966
		Black	3.109630^a^	.514499	.000	1.72293	4.49633
	Hispanic	White	−.918348	.659796	1.000	−2.69665	.85996
		Asian	−2.192963^a^	.514499	.000	− 3.57966	−.80627
		Black	.916667	.531944	.529	−.51705	2.35038
	Black	White	−1.835014^a^	.659796	.039	− 3.61332	−.05671
		Asian	−3.109630^a^	.514499	.000	− 4.49633	− 1.72293
		Hispanic	−.916667	.531944	.529	−2.35038	.51705
L3 TPA- Avg	White	Asian	−1.678251	.663278	.078	−3.46594	.10944
		Hispanic	.260513	.677639	1.000	−1.56589	2.08691
		Black	1.229957	.677639	.436	−.59644	3.05636
	Asian	White	1.678251	.663278	.078	−.10944	3.46594
		Hispanic	1.938763^a^	.528413	.002	.51457	3.36296
		Black	2.908208^a^	.528413	.000	1.48401	4.33241
	Hispanic	White	−.260513	.677639	1.000	−2.08691	1.56589
		Asian	−1.938763^a^	.528413	.002	− 3.36296	−.51457
		Black	.969444	.546330	.475	−.50305	2.44193
	Black	White	−1.229957	.677639	.436	−3.05636	.59644
		Asian	−2.908208^a^	.528413	.000	− 4.33241	− 1.48401
		Hispanic	−.969444	.546330	.475	−2.44193	.50305
L4 TPA- Avg	White	Asian	.3619231	.8285395	1.000	−1.871189	2.595035
		Hispanic	.6011823	.8464783	1.000	−1.680279	2.882643
		Black	1.9687749	.8464783	.133	−.312686	4.250236
	Asian	White	−.3619231	.8285395	1.000	−2.595035	1.871189
		Hispanic	.2392593	.6600710	1.000	−1.539790	2.018308
		Black	1.6068519	.6600710	.101	−.172197	3.385901
	Hispanic	White	−.6011823	.8464783	1.000	−2.882643	1.680279
		Asian	−.2392593	.6600710	1.000	−2.018308	1.539790
		Black	1.3675926	.6824526	.288	−.471780	3.206965
	Black	White	−1.9687749	.8464783	.133	−4.250236	.312686
		Asian	−1.6068519	.6600710	.101	−3.385901	.172197
		Hispanic	−1.3675926	.6824526	.288	−3.206965	.471780
L5 TPA- Avg	White	Asian	1.507134	.886641	.555	−.88258	3.89684
		Hispanic	.977749	.905838	1.000	−1.46370	3.41920
		Black	2.082194	.905838	.142	−.35926	4.52364
	Asian	White	−1.507134	.886641	.555	−3.89684	.88258
		Hispanic	−.529385	.706359	1.000	−2.43319	1.37442
		Black	.575060	.706359	1.000	−1.32875	2.47887
	Hispanic	White	−.977749	.905838	1.000	−3.41920	1.46370
		Asian	.529385	.706359	1.000	−1.37442	2.43319
		Black	1.104444	.730310	.803	−.86392	3.07280
	Black	White	−2.082194	.905838	.142	−4.52364	.35926
		Asian	−.575060	.706359	1.000	−2.47887	1.32875
		Hispanic	−1.104444	.730310	.803	−3.07280	.86392

^a^The mean difference is significant at the 0.05 level

## Discussion

Spinal surgery has been depicted to offer multiple benefits to patients. However, there still lacks an in-depth anatomical understanding of a key operative parameter, the TPA. The TPA is of great importance regarding optimal pedicle screw placement. We propose that furthering the understanding of morphological variation in the TPA will assist surgeons in preoperative planning of posterior lumbar fusion. In the current study, we have compared multiple patient factors with TPA including age, height, weight, gender and ethnicity. This study stands do delineate potential significant correlations between TPA and various patient demographics.

We did not find any statistically significant correlations between TPA in L1-L5 when compared to age, height, weight or gender. However, we did find statistically significant relationships between ethnicity and TPA. Here, we present a previously unknown relationship between the TPA of L2 and L3 and ethnicity. Specifically, at vertebral level L2, we have identified that the average TPA of Asian individuals is 3.11^o^ larger than that of Black individuals (*p* < 0.0001) and the TPA of White individuals is 1.84^o^larger than that of Black individuals (*p* = 0.039). At L3, we identified that the average TPA of Asian individuals is 1.94^o^ (*p* = 0.002) and 2.91^o^ (*p* < 0.0001) larger than that of Hispanic and Black individuals, respectively.

The results from this study are of potential value to the orthopedic surgeon performing posterior lumbar fusion techniques in both the preoperative planning stages and intraoperatively. When preparing for instrumentation of the lumbar spine, it is ideal to obtain a CT scan of the lumbar spine to evaluate for possible TPA variation. However, the utilization of CT imaging is not always undertaken in preoperative planning and may expose the patient to undue radiation. Knowing the variation in a given patient ethnicity prior to surgery may lead to faster operative times and more precise pedicle instrumentation leading to lower hardware failure rates and improved patient outcomes.

We had the advantage of utilizing CT images from a culturally diverse geographic area served by multiple hospitals. The CT scans evaluated were from living patient without complaints specific to lumbar spine pathology. To further validity of our study, the TPA data was obtained by a single observer and verified by 2 more senior physicians, enhancing interobserver variations.

Limitations of this study include lacking a standardized position of the patient on the CT scanner. Simpson et al. described inaccuracies even by CT when only evaluating on one plane, further emphasizing the need for careful preoperative evaluation of pedicle diameter [17]. Additionally, as race was self-reported with preset options, there was no ability to report multiple races. Finally, for White ethnicity, only 12 patients were included which may be unrepresentative of this population. Future work may be designed to address these limitations.

## Conclusion

We have identified a previously unknown and significant result with respect to TPA and ethnicity. At L2, individuals of Asian ethnicity were found to have larger TPAs than Black individuals and White individuals have larger TPA than Blacks. At L3, Asians were found to have a larger TPA than both Blacks and Hispanics. This result may help guide the orthopedic surgeon when preparing for lumbar instrumentation or any lumbar surgical techniques that require in depth knowledge of lumbar pedicle morphology. Further studies may be done to assess operative duration and clinical outcomes of surgeons who use this information in preoperative planning as well as intraoperative decision making compared to surgeons who are unaware of this ethnicity-TPA relationship.

## Abbreviations

CT: Computed Tomography
SD: Standard Deviation
SEM: Standard Error of the Mean
TPA: Transverse Pedicle Angle
